# Polyphenol Extract of *Moringa Oleifera* Leaves Alleviates Colonic Inflammation in Dextran Sulfate Sodium-Treated Mice

**DOI:** 10.1155/2020/6295402

**Published:** 2020-11-24

**Authors:** Yunjuan Zhang, Lei Peng, Wenyun Li, Tianyi Dai, Long Nie, Jing Xie, Yu Ai, Lingfei Li, Yang Tian, Jun Sheng

**Affiliations:** ^1^College of Food Science and Technology, Yunnan Agricultural University, Kunming 650201, China; ^2^Yunnan Provincial Key Laboratory of Biological Big Data, Yunnan Agricultural University, Kunming 650201, China; ^3^National R&D Center for Moringa Processing Technology, Yunnan Agricultural University, Kunming 650201, China; ^4^Key Laboratory of Pu-er Tea Science, Ministry of Education, Yunnan Agricultural University, Kunming 650224, China

## Abstract

*Moringa oleifera* Lam. is an essential herb used for the treatment of inflammation, diabetes, high blood pressure, and other diseases. In this study, phenolic extracts of *M. oleifera* leaves were obtained and analyzed. The results showed that the main identifiable phenols were astragalin, chlorogenic acid, isoquercitrin, kaempferitrin, luteolin, quercetin, and rutin. The effects of *M. oleifera* polyphenol extract (MOPE) on experimental colitis induced by 3% dextran sulfate sodium (DSS) were investigated. The results showed that oral administration of MOPE significantly alleviated the symptoms of DSS-induced colitis. MOPE significantly reduced weight loss, the disease activity index, colon shortening, and mucosal damage. In addition, MOPE attenuated the infiltration of CD3^+^ T cells, CD177^+^ neutrophils, and F4/80^+^ macrophages and significantly inhibited the secretion of IL-6 and TNF-*α*. After the MOPE administration, the expression of proteins associated with the NF-*κ*B signaling pathway changed. Specifically, compared with that of the DSS group, the protein expression of NF-*κ*B *p65* and p-I*κ*B*α* was downregulated, and the expression of I*κ*B*α* was upregulated. This study revealed the anti-inflammatory effects and mechanisms of MOPE in the colon, indicating its potential use in preventing inflammation-driven diseases.

## 1. Introduction

*Moringa oleifera* Lam. has been historically used as nutritious food and traditional medicine in China [1]. In 2012, *M. oleifera* leaves were approved by the Chinese Ministry of Health as a new resource food. *M. oleifera* is also an essential traditional herb used to treat inflammation, diabetes, hypertension, anemia, hypoimmunity, and other diseases [2–5]. There are some flavonoid pigments, such as alkaloids, kaempferol, rhamnose, and isoquercitrin, and various antioxidant compounds, such as ascorbic acid, flavonoids, phenols, and carotenoids, which occur naturally in *M. oleifera* [6] and may contribute to the anti-inflammatory activity of *M. oleifera*. Additionally, it has been previously reported that some active components in *M. oleifera* mitigate some chronic inflammation [7]. However, there have been few reports on the improvement and alleviation of inflammation by *M. oleifera* polyphenols.

Inflammatory bowel disease (IBD), including ulcerative colitis (UC) and Crohn's disease (CD) [8], is a chronic recurrent disease of the intestine [9]. IBD is exceptionally harmful and is associated with an increased risk for colon cancer [10]. IBD is affected by diets such as high-fat diets, and the incidence is high in some developed countries, such as Europe [8]. Recently, the incidence of IBD in Asia has increased rapidly due to the improvements in living conditions and the adoption of a Western lifestyle. The pathogenesis of IBD is not yet fully clear; it is generally considered to be related to the environment, infection, genetics, psychology, and immunity. In addition, the intestinal mucosal immune system plays an essential role in the pathogenesis of IBD [11]. Several studies have shown that there is a high level of activated transcription factor-kappa B in the intestinal mucosa of IBD patients, and nuclear transcription factor kappa B (NF-*κ*B) is considered to be one of the most critical signaling pathways in IBD. High expression of NF-*κ*B can enhance the release of proinflammatory cytokines, such as tumor necrosis factor-*α* (TNF-*α*) and interleukin-6 (IL-6), which leads to colonic tissue damage [12]. It has been suggested that the activation of NF-*κ*B plays a crucial role in the regulation of immune and inflammatory responses, and thus inhibiting the NF-*κ*B signaling pathway is a novel therapeutic strategy in IBD treatment research.

To date, there are no ideal drugs for the treatment of IBD. Existing treatments mainly include resection and relief medication, such as 5-aminosalicylic (5-ASA), corticosteroids, immunomodulatory drugs, and biological agents. However, long-term administration of these drugs has various side effects, including drug resistance and drug tolerance [13]. Therefore, it is necessary to find natural treatments with fewer side effects. Although *M. oleifera* has been reported to exhibit various bioactivities, knowledge about the effects of *M. oleifera* polyphenol extract (MOPE) on gut health is limited. The present study aimed to evaluate the effects of MOPE in an established model of acute ulcerative colitis induced with dextran sulfate sodium (DSS) and further explore its underlying mechanisms.

## 2. Materials and Methods

### 2.1. Preparation of MOPE

Dried *M. oleifera* leaves (Yunnan Shenbaofu Technology Development Co., Ltd., Dehong, China) were crushed and then extracted by the ultrasound-assisted method as described by Fei et al. [14]. The extraction process was performed three times under the same conditions with 70% ethanol as the solvent, a solid/solvent ratio of 1/30, and 250 W of ultrasonic power for 20 min. These three extraction supernatants were combined and passed through a D101 macroporous resin, followed by elution with an 80% ethanol solution to obtain an ethanol eluate. The eluate was vacuum freeze-dried to obtain the MOPE.

### 2.2. Ultrahigh-Performance Liquid Chromatography Quadrupole Time-of-Flight Tandem Mass Spectrometry (UPLC-QTOF-MS/MS)

The composition of the MOPE was analyzed using a UPLC-QTOF-MS/MS system (XEVO G2-S QTOF-MS, Waters, USA). The chromatographic conditions were as follows: ACQUITY UPLC HSS T3 C18 (2.1 × 100 mm, 1.8 *μ*m); mobile phase A (0.1% formic acid-water), mobile phase B (acetonitrile); flow rate: 0.5 mL·min^−1^; column temperature: 40°C; and analysis time 13 min. The sample was dissolved in 50% aqueous methanol, and the supernatant was used for analysis. The gradient elution was carried out as follows: 0 min, 10% B; 6 min, 40% B; 6.2 min, 60% B; 8.5 min, 90% B; 10.5 min, 90% B; and 10.6 min, 10% B. The mass spectrometry conditions were as follows: ESI: negative ion mode; scan range: 100–1200 Da; source temperature: 100°C; desolvation temperature: 350°C; desolvation gas flow: 700 L·h^−1^; LockSpray: leucine enkephalin (5-Leucine) enkephalin (LE); capillary: 2.8 kV; and sampling cone: 40 V.

### 2.3. Animal Treatments

The animal experiments were performed according to international guidelines. The protocol was approved by the Animal Care and Use Committee of Yunnan Agricultural University (No. YNAU-2017-011). Six-week-old male C57BL/6 mice (18–20 g, Liaoning Changsheng Biotechnology Co. Ltd., Liaoning, China) were maintained on a 12/12 h light/dark cycle at 25 ± 1°C and 55% humidity. All mice were housed 5/cage and had free access to standard mouse feed and tap water. The mice were allowed to acclimate for one week before the study began.

### 2.4. Induction of Colitis and MOPE Treatment

**Figure 1:**
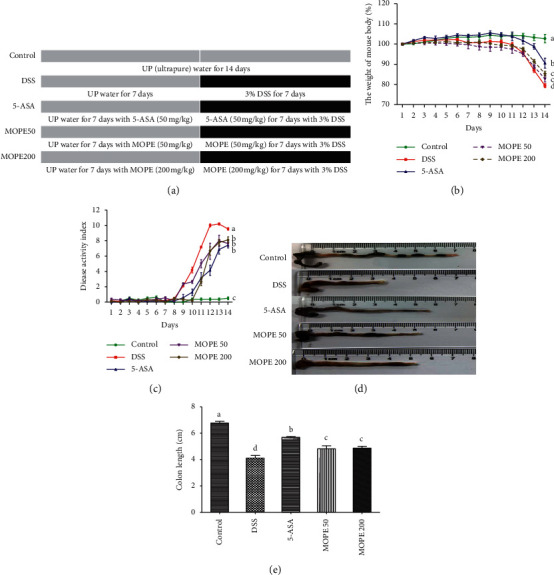
MOPE ameliorated DSS-induced colitis symptoms in mice. (a) The experimental design (*n* = 10 per group). (b) Body weight change in mice. (c) Disease activity index (DAI) scores. (d) Representative images of the mouse colon. (e) Colon lengths. The data are presented as the mean ± SEM. Values with different letters differ significantly (*p* < 0.05).

Colitis was induced with dextran sulfate sodium (DSS, 36000–50000, MP Biomedicals, USA, 2160110). The mice were randomly divided into five groups (*n* = 10 per group): the control group was given ultrapure (UP) water for 14 days; the DSS group was given UP water for the first seven days and then 3% DSS for the following seven days; the 5-aminosalicylic acid (5-ASA, positive control) group and the MOPE groups (50 mg/kg and 200 mg/kg, respectively) were given 5-ASA and MOPE individually for 14 days and were simultaneously given the water containing 3% DSS starting on the eighth day (Figure 1(a)).

### 2.5. Evaluation of the Disease Activity Index (DAI)

Mice were monitored daily for the development of colitis based on body weight changes, gross rectal bleeding, and stool consistency. The disease activity index (DAI) scores were measured for each animal according to a previously described method [15]. The details of the DAI grading standards are listed in the Supplementary Materials (Table S1).

### 2.6. Histopathology

The colon tissues 1 cm from the distal colon were rinsed with ice-cold PBS, fixed in 10% (v/v) neutral formalin, embedded in paraffin, cut into 5 µm slices (Leica, RM2126RT, Germany), and stained with hematoxylin and eosin (H&E). Histological analysis was performed according to previously described methods [16]. A detailed description of the score standards is listed in the Supplementary Materials (Table S2).

### 2.7. Immunohistochemistry

The tissue slices were deparaffinized by xylene for 0.5–1 h and rehydrated sequentially with 100%, 100%, 95%, and 80% ethanol and water for 5 min each. Then, the slices were immersed in methanol containing 3% hydrogen peroxide, followed by high-pressure antigen repair. The slides were incubated with primary antibodies against CD3^+^, CD177^+^, and F4/80^+^ (Abcam, Cambridge, MA) overnight and then incubated with the appropriate secondary antibodies, followed by diaminobenzidine (DAB) color development, hematoxylin staining, and xylene clearing. Immunostained tissue slices were visualized under a microscope (Olympus, Japan).

### 2.8. Determination of Serum Inflammatory Factors

At the end of the experiment, serum samples were prepared and subjected to enzyme-linked immunosorbent assay (ELISA) to determine serum levels of IL-6 and TNF-*α* using ELISA kits from BD Pharmingen. The procedure was performed according to the instructions.

### 2.9. Western Blotting

The colon tissues were homogenized and lysed in RIPA buffer (Solarbio) containing PMSF (100 : 1) for 15 min. After centrifugation (4°C, 15000 rpm, 10 min), the supernatant was collected, and the protein concentration was quantified using BCA protein assay reagent (Beyotime). The protein lysates (50 *μ*g protein) were separated by 10% sodium dodecyl sulfate-polyacrylamide gel electrophoresis and transferred to PVDF membranes (Immobilon). After being blocked with 5% bovine serum albumin (BSA) solution, the membranes were incubated with primary antibodies against TNF-*α*, NF-*κ*B *p65*, I*κ*B*α*, and phosphorylated I*κ*B*α*, followed by the appropriate secondary antibodies. Then, the protein-antibody complexes were developed with ECL Luminol reagent (Santa Cruz Biotechnology) and visualized.

### 2.10. Statistical Analysis

GraphPad Prism software version 5.0 was used to graph the results. The data are presented as the mean ± SEM. Statistical analysis was performed by SPSS 19.0. The differences between multiple groups were analyzed by one-way analysis of variance (ANOVA), followed by Duncan's test. The results were considered statistically significant when *p* < 0.05.

## 3. Results

### 3.1. UPLC-QTOF-MS/MS Analysis of MOPE

**Figure 2:**
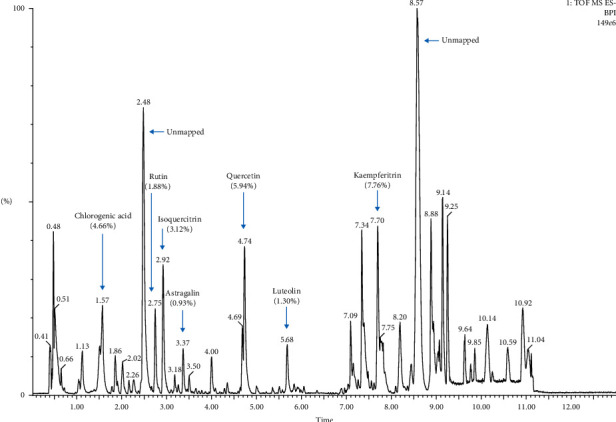
UPLC-QTOF-MS/MS chromatogram of MOPE.

Seven compounds, including chlorogenic acid, rutin, isoquercitrin, astragalin, quercetin, luteolin, and kaempferitrin, which were known phenolic substances from MOPE, were analyzed by UPLC-QTOF-MS/MS. The retention times of these seven compounds were 1.57, 2.75, 2.92, 3.37, 4.74, 5.68, and 7.70 min, respectively. The relative quantities were 4.66%, 1.88%, 3.12%, 0.93%, 5.94%, 1.30%, and 7.76%, respectively (Figure 2).

### 3.2. MOPE Ameliorated Colitis Symptoms in Mice

The mice were treated with a low (50 mg/kg) or a high dose (200 mg/kg) of MOPE for 2 weeks (Figure 1(a)). Bodyweights and DAI scores of the mice were monitored every day throughout the experiment. The DSS-only treatment group exhibited severe bodyweight loss, while treatment with 5-ASA (positive control) and 50 mg/kg and 200 mg/kg MOPE significantly alleviated weight loss (Figure 1(b)). Additionally, mice in the DSS group exhibited high DAI scores, while 5-ASA and MOPE treatment significantly decreased the DAI scores compared to those of the DSS group (Figure 1(c)). These results indicated that 5-ASA and MOPE treatments alleviated colonic inflammatory symptoms in mice.

The length of the colon is an important indicator for indirectly assessing the severity of colitis. In this study, fecal residues in the colons of control mice were normal and granular. The DSS group was induced to develop colitis, exhibiting hyperemic colons and very severe colon shortening. Furthermore, fecal residue in the colon in the 5-ASA group and MOPE groups was loose and less hyperemic (Figure 1(d)). 5-ASA and MOPE treatment significantly prevented the colonic shortening compared to that of the DSS-treated group (Figure 1(e)).

### 3.3. MOPE Ameliorated Colonic Injury in Mice with DSS-Induced Colitis

**Figure 3:**
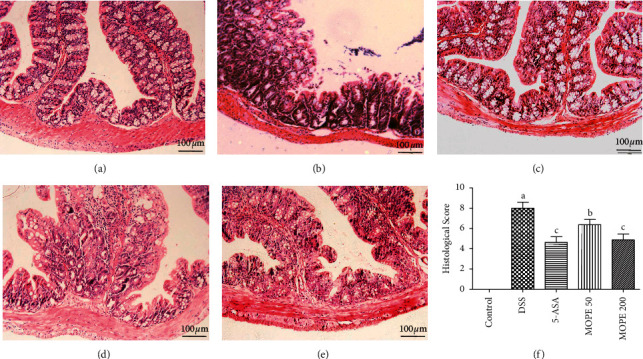
Effects of MOPE on the histopathological characterization of DSS-colitis mice. Representative HE-stained sections of the distal colonic tissues from the (a) control, (b) DSS, (c) 5-ASA (50 mg/kg), (d) MOPE (50 mg/kg), and (e) MOPE (200 mg/kg) groups. All images were acquired using 200× magnification. (f) Histological scores of colonic abnormalities. Values with different letters (a-c) differ significantly (*p* < 0.05).

The histological examination of colonic tissue from healthy mice revealed that the epithelial cells and crypts were structurally intact, the glands were arranged neatly, and there was no inflammatory cell infiltration (Figure 3(a)). In contrast, in the DSS-treated group, the lamina propria was damaged, the glands were destroyed and arranged irregularly, the epithelial cells and crypt structures were destroyed, and a large number of inflammatory cells infiltrated the muscle floor (Figure 3(b)). This damage was significantly alleviated after treatment with 5-ASA and MOPE (Figures 3(c)–3(f)).

### 3.4. MOPE Attenuated the Infiltration of Inflammatory Cells in Mice with DSS-Induced Colitis

**Figure 4:**
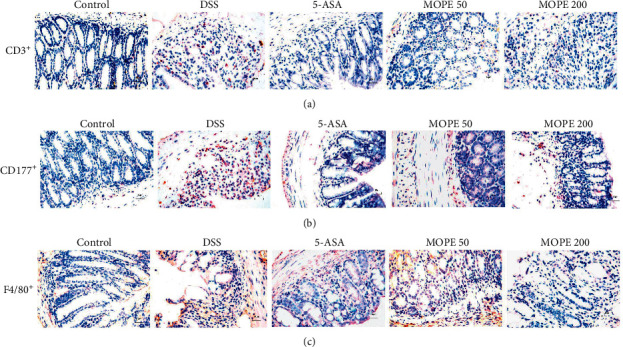
MOPE attenuated the infiltration of inflammatory cells in mice with DSS-induced colitis. Representative images of (a) CD3^+^, (b) CD177^+^, (c) and F4/80^+^ immunostaining in the distal colons of mice; original magnification: 400×.

The expression of CD3^+^, CD177^+^, and F4/80^+^ in the distal colon was measured by immunohistochemistry to detect T cell, neutrophil, and macrophage infiltration, respectively. The results indicated that T cells, neutrophils, and macrophages were increased in DSS-treated mice compared to normal mice, indicating that these inflammatory cells infiltrated the colonic injury area. 5-ASA and MOPE treatment reduced the infiltration of T cells, neutrophils, and macrophages (Figure 4).

### 3.5. MOPE Decreased the Cytokine Levels in Mice with DSS-Induced Colitis

**Figure 5:**
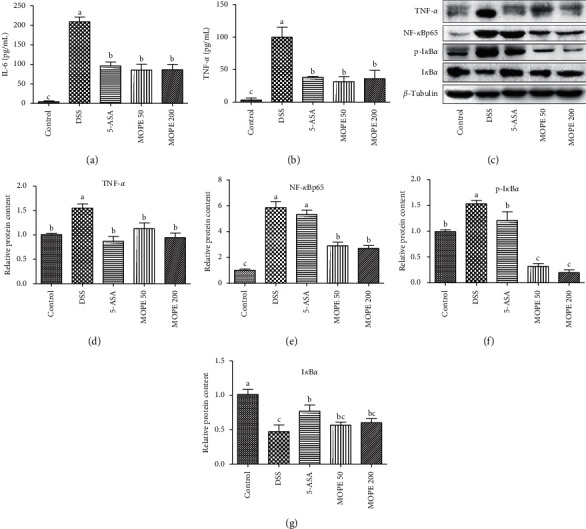
MOPE modulated cytokines and inflammation-related signaling proteins in mice with DSS-induced colitis. (a) The serum IL-6 level. (b) The serum TNF-*α*. (c) Western blot analysis of key signaling proteins in colonic tissue. (d) Quantitative analysis of TNF-*α* protein levels in colonic tissue. (e) Quantitative analysis of NF-*κ*B *p65* protein levels. (f) Quantitative analysis of (p)-I*κ*B*α* protein levels. (g) Quantitative analysis of I*κ*B*α* protein levels. Values with different letters (a-c) differ significantly (*p* < 0.05).

IL-6 and TNF-*α* are two typical inflammatory cytokines. The serum levels of IL-6 and TNF-*α* were measured in the present study. Compared with those of the control group, substantial increases in IL-6 (Figure 5(a)) and TNF-*α* (Figure 5(b)) levels were observed in the DSS group (*p* < 0.001). However, MOPE treatment significantly reduced the serum levels of IL-6 (*p* < 0.01) and TNF-*α* (*p* < 0.05), and the effect was the same as that of 5-ASA (positive control). Moreover, MOPE treatment significantly inhibited the protein expression of TNF-*α* in colon tissue after DSS-induced colitis (Figures 5(c) and 5(d)).

### 3.6. MOPE Modulated Inflammation-Related Signaling Proteins

The NF-*κ*B signaling pathway strongly influences the pathogenesis of colitis. In the present study, the protein expression of NF-*κ*B *p65* in the nucleus and p-I*κ*B*α* in the colon tissue of DSS model mice was higher than that of control mice, and the protein expression of I*κ*B*α* was decreased (Figures 5(d)–5(g)). This result indicated that the NF-*κ*B signaling pathway was activated in the colonic mucosal. After treatment with MOPE, the protein expression of NF-*κ*B *p65* and p-I*κ*B*α* was significantly downregulated, and the expression of I*κ*B*α* was upregulated. These results suggested that the anti-inflammatory effects of MOPE were related to inhibiting NF-*κ*B pathway activation.

## 4. Discussion

*M. oleifera* has been reported to have beneficial effects on various diseases, including diabetes, hypertension, hyperlipidemia, and other chronic inflammation. There are some individual bioactive components in *M. oleifera* extracts that have potential preventive and therapeutic effects on inflammation [17]. Studies have shown that extracts of *M. oleifera* inhibit the production of NO and proinflammatory cytokines in LPS-induced macrophages [7, 18]. The pods of *M. oleifera* can inhibit the LPS-induced expression of IL-6 and TNF-*α* and inhibit LPS-induced I*κ*B activation [19]. The ethyl acetate extract of *M. oleifera* can significantly inhibit LPS-induced TNF-*α*, IL-6, and IL-8 production in human monocyte-derived macrophages (MDMs). *M. oleifera* extracts effectively inhibit the expression of inflammatory mediators that may be related to the inhibition of the NF-*κ*B signaling pathway [3, 20]. In addition, it has been reported that the hydroalcoholic extract of *M. oleifera* seeds can reduce acetic acid-induced colitis in rats [21]. Moreover, the isothiocyanate extracted from *M. oleifera* seeds can effectively alleviate DSS-induced colitis [22]. However, knowledge about the effects of MOPE on gut health is limited. Therefore, the present study aimed to investigate MOPE-mediated alleviation of DSS-induced colitis in mice.

Here, we showed that MOPE could significantly ameliorate the symptoms of DSS-induced colitis, including mitigating body weight loss, colonic tissue damage, the secretion of inflammatory cytokines, and the infiltration of inflammatory cells. IL-6 and TNF-*α* are proinflammatory cytokines that mediate inflammatory responses in the UC model. Excessive secretion of these inflammatory cytokines can cause colitis [15]. Many studies have reported that the levels of IL-6 and TNF-*α* in IBD mice are increased [23–25]. IL-6 is an interleukin produced by a variety of cells and is closely related to inflammation and the immune response. IL-6 stimulates neutrophil chemotaxis and causes tissue destruction in the colon. Some studies have found that IL-6 is elevated in many IBDs and may be associated with the NF-*κ*B signaling pathway [26, 27]. TNF-*α*, a cytokine with multiple effects that is produced by activated T cells, plays an essential role in the pathogenesis of colitis by triggering the accumulation and activation of leukocytes. TNF-*α* overexpression is vital for the pathogenesis of the intestinal mucosa [28, 29]. In the present study, DSS induced an increase in TNF-*α* and IL-6 in mice, whereas MOPE treatment significantly reduced the increases in both inflammatory factors.

NF-*κ*B is a classic signaling pathway associated with inflammation. Several studies have shown that some natural compounds, such as caffeic acid, blueberry polyphenols, and *Aster glehni* extract, can improve UC by inhibiting the NF-*κ*B pathway [25, 30–32]. It was shown that the ethyl acetate extract of *M. oleifera* inhibited NF-*κ*B p65 in RAW 264.7 cells, and it exerted anti-inflammatory effects by upregulating the expression of I*κ*B inhibitor (I*κ*B*α*) and blocking the nuclear translocation of NF-*κ*B [3]. NF-*κ*B family members are retained in the cytoplasm bound to a class of inhibitory proteins termed I*κ*Bs [33]. When cells are activated, I*κ*B is phosphorylated by I*κ*B kinase (IKK) and degraded, after which the released NF-*κ*B translocates to the nucleus and activates transcription. In other words, phosphorylation of I*κ*B (p-I*κ*B) is a crucial step in NF-*κ*B activation, which leads to activation of the NF-*κ*B signaling pathway [34]. The increase in TNF-*α*-related indicators is an essential manifestation of NF-*κ*B pathway activation. Many treatments target TNF-*α*, and some related drugs, including TNF-*α* blockers, have been successfully used in the treatment of patients with IBD [31, 35]. For these reasons, the proteins related to the NF-*κ*B signaling pathways were investigated in the present study. Our results showed that the protein expression of NF-*κ*B *p65* in the nucleus and p-I*κ*B*α* and TNF-*α* in colon tissue of DSS model mice was higher than that of control mice, and the expression of I*κ*B*α* was decreased. This result indicated that the NF-*κ*B pathway was activated in the colonic mucosa. After MOPE administration, the protein expression of NF-*κ*B *p65* and p-I*κ*B*α* was downregulated, and the expression of I*κ*B*α* was upregulated. Furthermore, MOPE treatment inhibited the high expression of TNF-*α* in colon tissue. These results indicate that the anti-inflammatory effects of MOPE may be related to the inhibition of the NF-*κ*B signaling pathway.

In the present study, MOPE is a mixed extract. The main phenolic substances in MOPE are kaempferol, quercetin, chlorogenic acid, isoquercitrin, rutin, and luteolin, as determined by UPLC-QTOF-MS/MS analysis, which is consistent with the results of the study by Zhu et al. [36]. In the future, we will focus on the separation and purification of *M. oleifera* polyphenols to determine which phenolic substance has anti-inflammatory or multicomponent synergistic effects.

In conclusion, our research shows that MOPE can alleviate DSS-induced colitis, including mitigating body weight loss, colon shortening, the secretion of inflammatory cytokines, colon tissue damage, and inflammatory cell infiltration. Further study examination shows that MOPE may alleviate colitis by inhibiting NF-*κ*B signaling pathway activation. These results indicate that *M. oleifera* can be developed as a potential health food for preventing colitis.

## Data Availability

The data to support the findings of this study are included within the article. Other data used to support the findings of this study are available from the corresponding author upon request.

## Supplementary Materials

Supplementary Materials The details of the DAI grading standards are listed in the Supplementary Materials (Table S1). A detailed description of the Histological Scores of Colon Damage is listed in the Supplementary Materials (Table S2).
