# Supplementary material for: Polyphenol Extract of Moringa Oleifera Leaves Alleviates Colonic Inflammation in Dextran Sulfate Sodium-Treated Mice
Source: Evid Based Complement Alternat Med. 2020 Nov 24;2020:6295402. doi: 10.1155/2020/6295402 (PMC7710425; doi:10.1155/2020/6295402)
Supplement: Supplementary Materials — The details of the DAI grading standards are listed in the Supplementary Materials (Table S1). A detailed description of the Histological Scores of Colon Damage is listed in the Supplementary Materials (Table S2). [file 6295402.f1.doc]

T_ABLE_ S1: Criteria for disease activity index (DAI)

| Weight loss(%) | Stool Consistency | Bloodstain or gross bleeding | Score |
| --- | --- | --- | --- |
| None | Normal | Negative | 0 |
| 1-5 | Loose stool | Negative | 1 |
| 5-10 | Loose stool | Positive | 2 |
| 10-15 | Diarrhea | Positive | 3 |
| ＞15 | Diarrhea | Gross bleeding | 4 |

T_ABLE_ S2: Histological Scores of Colon Damage

| Score | Inflammation severity | Inflammation extent | Crypt damage |
| --- | --- | --- | --- |
| 0 | None | None | None |
| 1 | Mild | Mucosa | Basal 1/3 damaged |
| 2 | Moderate | Mucosa and submucosa | Basal 2/3 damaged |
| 3 | Severe | Transmural | Crypts lost, surface epithelium present |
| 4 | - | - | Crypts and surface epithelium lost |
